# MRI and PET Image Fusion Using Fuzzy Logic and Image Local Features

**DOI:** 10.1155/2014/708075

**Published:** 2014-01-19

**Authors:** Umer Javed, Muhammad Mohsin Riaz, Abdul Ghafoor, Syed Sohaib Ali, Tanveer Ahmed Cheema

**Affiliations:** ^1^Faculty of Engineering and Technology, International Islamic University, Islamabad 44000, Pakistan; ^2^School of Engineering and Applied Sciences, Isra University, Islamabad 44000, Pakistan; ^3^College of Signals, National University of Sciences and Technology, Islamabad 44000, Pakistan

## Abstract

An image fusion technique for magnetic resonance imaging (MRI) and positron emission tomography (PET) using local features and fuzzy logic is presented. The aim of proposed technique is to maximally combine useful information present in MRI and PET images. Image local features are extracted and combined with fuzzy logic to compute weights for each pixel. Simulation results show that the proposed scheme produces significantly better results compared to state-of-art schemes.

## 1. Introduction

Fusion of images obtained from different imaging systems like computed tomography (CT), MRI, and PET plays an important role in medical diagnosis and other clinical applications. Each imaging technique provides a different level of information. For instance, CT (based on X-ray principle) is commonly used for visualizing dense structures and is not suitable for soft tissues and physiological analysis. MRI on the other hand provides better visualization of soft tissues and is commonly used for detection of tumors and other tissue abnormalities. Likewise, information of blood flow in the body is provided by PET (a nuclear imaging technique) but it suffers from low resolution as compared to CT and MRI. Hence, fusion of images obtained from different modalities is desirable to extract sufficient information for clinical diagnosis and treatment.

Image fusion integrates (complementary as well as redundant) information from multimodality images to create a fused image [1–6]. It not only provides accurate description of the same object but also helps in required memory reduction by storing fused images instead of multiple source images. Different techniques are developed for medical image fusion which can be generally grouped into pixel, feature, and decision level fusion [7]. Compared to feature and decision, pixel level methods [1, 2] are more suited for medical imaging as they can preserve spatial details in fused images [1, 8].

Conventional pixel level methods (including addition, subtraction, multiplication, and weighted average) are simpler but are less accurate. Intensity Hue saturation (IHS)-based methods fuse the images by replacing the intensity component [1, 5, 9]. These methods generally produce high-resolution fused images but cause spectral distortion (due to inaccurate estimation of spectral information) [10]. Similarly, principal components analysis based methods fuse images by replacing certain principle components [11].

Multiresolution techniques including pyramids, discrete wavelet transform (DWT), contourlet, curvelet, shearlet, and framelet transform image into different bands for fusion (a comprehensive comparison is presented in [12]). DWT-based schemes decompose the input images into horizontal, vertical, and diagonal subbands which are then fused using additive or substitutive methods. Earlier DWT-based fusion schemes cannot preserve the salient features of the source images efficiently, hence producing block artifacts and inconsistency in the fused results [2, 3]. Human visual system is combined with DWT to fuse the low frequency bands using visibility and variance features, respectively. Local window approach is used (to adjust coefficients adaptively) for noise reduction and maintaining homogeneity in fused image [4]. However, the method often produces block artifacts and reduced contrast [3, 5]. Consistency verification and activity measures combined with DWT can only capture limited directional information and hence are not suitable for sharp image transitions [13].

Texture features and visibility measure are used with framelet transform [5] to fuse high and low frequency components, respectively. Contourlet transform based methods use different and flexible directions to detect the intrinsic geometrical structures [13]. The common methods are variable weight using nonsubsampled contourlet transform [14]; and bio-inspired activity measurer using pulse-coded neural networks [15]. However, the down- and up-sampling in contourlet transform lack shift invariance and cause ringing artifacts [14]. Curvelet transform uses various directions and positions at length scales [16]; however, it does not provide a multiresolution representation of geometry [17]. Shearlet transform carries different features (like directionality, localization, and multiscale framework) and can decompose the image into any scale and direction to fuse the required information [17].

Prespecified transform matrix and learning techniques are used with kernel singular value decomposition to fuse images in sparse domain [18]. In [19], image fusion has been performed using redundancy DWT and contourlet transform. A pixel level neuro-fuzzy logic based fusion adjusts the membership functions (MFs) using backpropagation and least mean square algorithms [20]. A spiking cortical model is proposed to fuse different types of medical images [21]. However, these schemes are complex or work under certain assumptions/constraints.

A fusion technique for MRI and PET images using local features and fuzzy logic is presented. The proposed technique maximally combines the useful information present in MRI and PET images. Image local features are extracted and combined with fuzzy logic to compute weights for each pixel. Simulation results based on visual and quantitative analysis show the significance of the proposed scheme.

## 2. $\overset{`}{A}$-Trous-Based Image Fusion: An Overview

In contrast to conventional multiresolution schemes (where the output is downsampled after each level), *à*-trous or undecimated wavelet provides shift invariance, hence better suited for image fusion.

Let different approximations *I*_MRI,*k*_ of MRI image *I*_MRI_ (having dimensions *M* × *N*) be obtained by successive convolutions with a filter *f*, that is,

(1) $$\begin{array}{c} I_{\text{MRI},k+1}=I_{\text{MRI},k}*f, \end{array}$$

where *I*_MRI,0_ = *I*_MRI_ and *f* is a bicubic B-spline filter. The *k*th wavelet plane *W*_MRI,*k*_ of *I*_MRI_ is,

(2) $$\begin{array}{c} W_{\text{MRI},k}=I_{\text{MRI},k+1}-I_{\text{MRI},k}. \end{array}$$

The image *I*_MRI_ is decomposed into low *I*_MRI,*L*_ and high *I*_MRI,*H*_ frequency components as

(3) IMRI=IMRI,L+IMRI,H=IMRI,L+∑k=0KWMRI,k,

where *K* is the total number of decomposition levels. Similarly PET image *I*_PET_ in terms of low *I*_PET,*L*_ and high *I*_PET,*H*_ frequency components is

(4) $$\begin{array}{c} I_{\text{PET}}\left(\beta \right)=I_{\text{PET},L}\left(\beta \right)+\overset{K}{\underset{k=0}{\sum }}W_{\text{PET},k}\left(\beta \right), \end{array}$$

where *β* ∈ {*R*, *G*, *B*}, as PET images are assumed to be in pseudocolor [9].

Different methods are present in literature to fuse low and high frequency components which are generally grouped into substitute wavelet (SW) and additive wavelet (AW). The fused image *I*_SW_ using SW is

(5) $$\begin{array}{c} I_{\text{SW}}\left(\beta \right)=I_{\text{PET},L}\left(\beta \right)+\overset{K}{\underset{k=0}{\sum }}W_{\text{MRI},k}. \end{array}$$

Note that SW method fuses image by completely replacing the high frequency components of PET by high frequency components of MRI image, which can cause geometric and spectral distortion. SW and IHS (SWI) are combined to overcome the limitation in fused image *I*_SWI_; that is,

(6) $$\begin{array}{c} I_{\text{SWI}}\left(\beta \right)=I_{\text{PET},L}\left(\beta \right)-\overset{K}{\underset{k=0}{\sum }}W_{\text{INT},k}+\overset{K}{\underset{k=0}{\sum }}W_{\text{MRI},k}, \end{array}$$

where the intensity image *I*_INT_ is

(7) $$\begin{array}{c} I_{\text{INT}}=\frac{1}{B}\underset{\beta^{\prime }}{\sum }I_{\text{PET}}\left(\beta^{\prime }\right). \end{array}$$

The substitution process in SWI method sometimes results in loss of information as the intensity component is obtained by simple averaging/weighting.

In AW method, the fused image *I*_AW_ is obtained by injecting high frequency components of *I*_MRI_ into *I*_PET_:

(8) $$\begin{array}{c} I_{\text{AW}}\left(\beta \right)=I_{\text{PET}}\left(\beta \right)+\overset{K}{\underset{k=0}{\sum }}W_{\text{MRI},k}. \end{array}$$

AW method adds the same amount of high frequencies into low-resolution bands which causes redundancy of high frequency components (hence resulting in spectral distortion).

To cater the limitation, AW luminance proportional (AWLP) method injects the high frequencies in proportion to the intensity values [22]. Consider

(9) $$\begin{array}{c} I_{\text{AWLP}}\left(\beta \right)=I_{\text{PET}}\left(\beta \right)+\frac{I_{\text{PET}}\left(\beta \right)}{\left(1/B\right)\sum_{\beta^{\prime }}I_{\text{PET}}\left(\beta^{\prime }\right)}\overset{K}{\underset{k=1}{\sum }}W_{\text{MRI},k}, \end{array}$$

where *B* are total number of bands. The fused image *I*_AWLP_ of AWLP preserves the relative spectral information amongst different bands. The fused image using improved additive wavelet proportional (IAWP) [23] method is

(10) IIAWP(β)=IPET(β)+IPET(β)(1/B)∑β′IPET(β′),×[∑k=1KWMRI,k−∑k=1KWMRIR,k],

where *W*_MRIR,*k*_ are wavelet planes of a low-resolution (a spatially degraded version of *I*_MRI_) MRI image *I*_MRIR_. The *I*_MRIR_ is obtained by filtering the high frequencies (by applying a smoothing filter). The major limitations of the above schemes includes induction of redundant high/low frequencies; and consequently spatial degradations.

## 3. Proposed Technique

The proposed scheme first decomposes the MRI and PET images into low and high frequencies using *à*-trous wavelet. High and low frequencies are then fused separately according to defined criterion. The overall fused image *I*_*F*_ in terms of high *I*_*F*,*H*_ and low *I*_*F*,*L*_(*β*) frequencies is

(11) $$\begin{array}{c} I_{F}\left(\beta \right)=I_{F,L}\left(\beta \right)+I_{F,H}. \end{array}$$

### 3.1. Fusion of Low Frequencies

Fusion of low frequencies *I*_MRI,*L*_ and *I*_PET,*L*_ is critical and challenging task. Various schemes utilize different criterions for fusion of low frequencies. For instance, one choice is to totally discard the low frequencies of one image, another choice is to take average or weighted average of both and so forth. However, the schemes provide limited performance as they do not cater the spatial properties of image. We have proposed fusion of low frequency using different weighting average for each pixel location. The weights are computed based on the amount of information contained in vicinity of each pixel.

#### 3.1.1. Local Features

Local variance (LV) and local blur (LB) features are used with fuzzy inference engine to compute the desired weights for fusing low frequencies.

LV [24] is used to evaluate the regional characteristics of *I*_PET,*L*_ image and is defined as *I*_LV_:

(12) ILV(β,m,n)=1(2m1+1)(2n1+1)×∑m2=m−m1m+m1 ∑n2=n−n1n+n1(IPET(β,m2,n2)−I¯PET(β))2,

where ${\bar{I}}_{\text{PET}}(\beta )$ is the mean value of *m*_1_ × *n*_1_ window centered at (*m*, *n*) pixel. Note that image containing sharp edges results in higher value (and vice versa).

LB *I*_LB_ is computed using local Rényi entropy [25] of *I*_PET,*L*_ image. Let *P*_*βmn*_(*k*) be the probability (or normalized histogram) having intensity values *k* = 1,2,…, *K* within a local window (of size *m*_1_ × *n*_1_) centered at (*β*, *m*, *n*) pixel. *I*_LB_ is defined as [25]

(13) $$\begin{array}{c} I_{\text{LB}}\left(\beta ,m,n\right)=-\frac{1}{2}\ln\left(\overset{K}{\underset{k}{\sum }}P_{\beta mn}^{3}\left(k\right)\right). \end{array}$$

High values of *I*_LV_ and *I*_LB_ show that *I*_PET,*L*_ contain more information and need to be assigned more weight as compared to *I*_MRI,*L*_ image.

#### 3.1.2. Fuzzy Inference Engine

Let high *ζ*_LV,1_(*u*) and low *ζ*_LV,2_(*u*) Gaussian Membership functions (MFs) having means ${\bar{u}}^{\left(1\right)}$, ${\bar{u}}^{\left(2\right)}$ and variances *σ*_*u*_^(1)^, *σ*_*u*_^(2)^ for LV be [26]

(14) $$\begin{array}{c} \zeta_{\text{LV},1}\left(u\right)=e^{-{\left(\left(u-{\bar{u}}^{\left(1\right)}\right)/\sigma_{u}^{\left(1\right)}\right)}^{2}},\;\;\zeta_{\text{LV},2}\left(u\right)=e^{-{\left((u-{\bar{u}}^{\left(2\right)})/\sigma_{u}^{\left(2\right)}\right)}^{2}}. \end{array}$$

Similarly let high *ζ*_LB,1_(*v*) and low *μ*_LB,2_(*v*) Gaussian MFs having means ${\bar{v}}^{\left(1\right)}$, ${\bar{v}}^{\left(2\right)}$ and variances *σ*_*v*_^(1)^, *σ*_*v*_^(2)^ for LB be

(15) $$\begin{array}{c} \zeta_{\text{LB},L}\left(v\right)=e^{-{\left((v-{\bar{v}}^{L})/\sigma_{v}^{L}\right)}^{2}},\;\;\zeta_{\text{LB},H}\left(v\right)=e^{-{\left((v-{\bar{v}}^{H})/\sigma_{v}^{H}\right)}^{2}}. \end{array}$$

The inputs *I*_LV_(*β*, *m*, *n*) and *I*_LV_(*β*, *m*, *n*) are mapped into fuzzy set using Gaussian fuzzifier [27] as

(16) $$\begin{array}{c} \zeta_{\text{LV},\text{LB}}\left(u,v\right)=e^{-{\left((u-I_{\text{LV}}(\beta ,m,n))/\varsigma_{1}\right)}^{2}}\times e^{-{\left((v-I_{\text{LB}}(\beta ,m,n))/\varsigma_{2}\right)}^{2}}, \end{array}$$

where *ς*_1_ and *ς*_2_ are noise suppression parameters. The inputs are then processed by fuzzy inference engine using pre defined IF-THEN rules [26, 27] as follows.

 
*Ru* ^(1)^: IF *I*_LV_(*β*, *m*, *n*) is high and *I*_LB_(*β*, *m*, *n*) is high THEN *I*_WT_(*β*, *m*, *n*) is high.
 
*Ru* ^(2)^: IF *I*_LV_(*β*, *m*, *n*) is low and *I*_LB_(*β*, *m*, *n*) is high THEN *I*_WT_(*β*, *m*, *n*) is medium.
 
*Ru* ^(3)^: IF *I*_LV_(*β*, *m*, *n*) is high and *I*_LB_(*β*, *m*, *n*) is low,THEN *I*_WT_(*β*, *m*, *n*) is medium.
 
*Ru* ^(4)^: IF *I*_LV_(*β*, *m*, *n*) is low and *I*_LB_(*β*, *m*, *n*) is low THEN *I*_WT_(*β*, *m*, *n*) is low.

The output MFs for high (having mean ${\bar{y}}^{\left(1\right)}$ and variance *σ*_*y*_^(1)^), medium (having mean ${\bar{y}}^{\left(2\right)}$ and variance *σ*_*y*_^(2)^), and low (having mean ${\bar{y}}^{\left(3\right)}$ and variance *σ*_*y*_^(3)^) are defined as

(17) $$\begin{array}{c} \zeta_{W,1}\left(y\right)=e^{-{\left((y-{\bar{y}}^{\left(1\right)})/\sigma_{y}^{\left(1\right)}\right)}^{2}},\\ \zeta_{W,2}\left(y\right)=e^{-{\left((y-{\bar{y}}^{\left(2\right)})/\sigma_{y}^{\left(2\right)}\right)}^{2}},\;\;\zeta_{W,3}\left(y\right)=e^{-{\left((y-{\bar{y}}^{\left(3\right)})/\sigma_{y}^{\left(3\right)}\right)}^{2}}. \end{array}$$

The output of fuzzy inference engine is

(18) $$\begin{array}{c} \zeta_{W,L}^{\prime }\left(y\right)=\underset{\left\{c,d,e\right\}}{\max}\left[\underset{\left\{u,v\right\}}{\sup}\zeta_{\text{LV},\text{LB}}\left(u,v\right)\zeta_{\text{LV},c}\left(u\right)\zeta_{\text{LV},d}\left(v\right)\zeta_{W,e}\left(y\right)\right], \end{array}$$

where {*c*, *d*}∈{1,2} and *e* ∈ {1,2, 3}. The weights *I*_WT_(*β*, *m*, *n*) are obtained by processing fuzzy outputs using center average defuzzifier [27].

The *I*_*F*,*L*_(*β*) image is obtained by weighted sum of *I*_PET,*L*_ and *I*_MRI,*L*_ as

(19) IF,L(β,m,n)=IWT(β,m,n)IPET,L(β,m,n)+(1−IWT(β,m,n))IMRI,L(m,n).

### 3.2. Fusion of High Frequencies

Let *W*_MRI-MRIR,*k*_ represent a wavelet plane of the resultant image *I*_MRI_ − *I*_MRIR_. This ensures that only those high frequency components are used for image fusion, which are not already present in *I*_MRI_. By the virtue of this, the proposed scheme not only avoids redundancy of information but also results in improved fusion results as compared to early techniques. The fused high frequency image *I*_*F*,*H*_ is

(20) $$\begin{array}{c} I_{F,H}=\overset{K}{\underset{k=1}{\sum }}W_{\text{MRI-MRIR},k}. \end{array}$$

Note that *I*_*F*,*H*_ is not dependent on the bands *β* because *I*_MRI_ is gray-scale image.

## 4. Results and Discussion

The simulations of proposed and existing schemes are performed on PET and MRI images obtained from Harvard database [28]. The fusion database for brain images is classified into normal, grade II astrocytoma, and grade IV astrocytoma images. The MRI and PET images are coregistered with 256 × 256 spatial resolution. The proposed fusion scheme is compared visually and quantitatively (using entropy [29], mutual information (MI) [29], structural similarity (SSIM) [30], Xydeas and Petrovic [31] metric, and Piella [32] metric) with DWT [12], GIHS [6], IAWP [23], and GFF [33] schemes.

**Figure 1:**
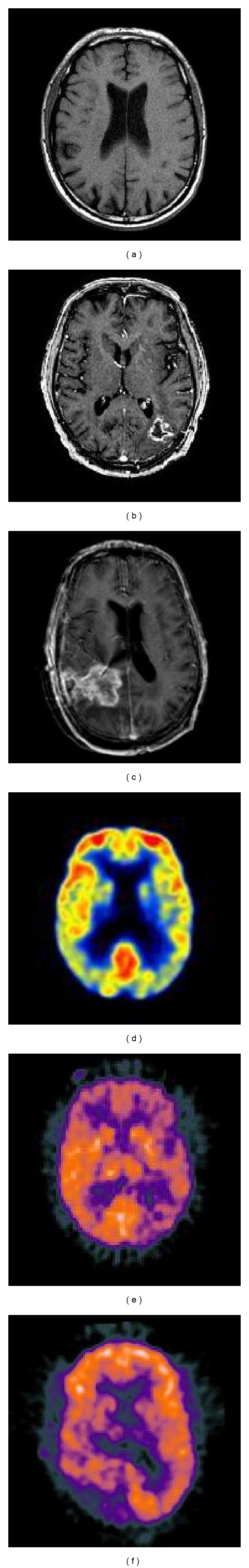
Original MRI and PET images: (a)–(c) MRI; (d)–(f) PET.

The original MRI images belonging to normal brain, grade II astrocytoma, and grade IV astrocytoma are shown in Figures 1(a)–1(c), respectively. Fluorodeoxyglucose (FDG) is a radiopharmaceutical commonly used for PET scans. The PET-FDG images of normal, grade II, and grade IV astrocytoma are shown in Figures 1(d)–1(f), respectively. It can be seen that different imaging modalities provide complementary information for the same region.

**Figure 2:**
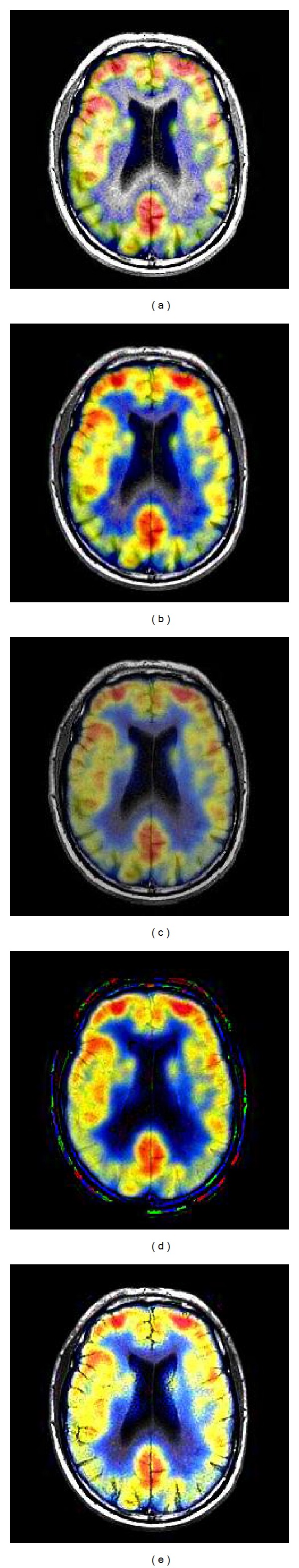
Image fusion results for normal images: (a) DWT [12]; (b) GIHS [6]; (c) GFF [33]; (d) IAWP [23]; (e) proposed technique.

Figure 2 shows fused images (of normal brain) obtained by using different techniques. It can be seen from Figure 2(e) that the proposed technique has preserved the complementary information of both modalities and the fuzzy based weight assessment has enabled offering less spectral information loss as compared to other state-of-art techniques.

**Figure 3:**
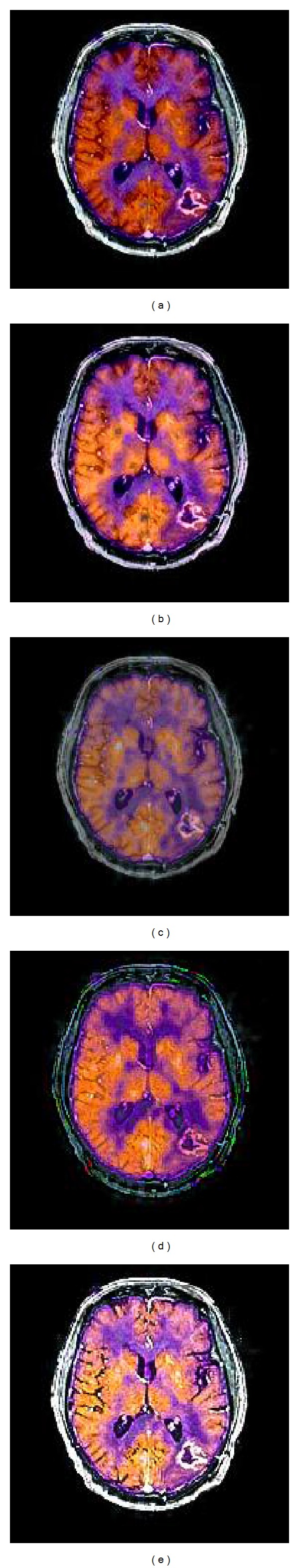
Image fusion results for grade II astrocytoma images: (a) DWT [12]; (b) GIHS [6]; (c) GFF [33]; (d) IAWP [23]; (e) proposed technique.

Figure 3 shows fused images (of grade II astrocytoma class) obtained by using different techniques. From Figure 3(e), it can be observed that the proposed technique provides complementary information contained in both modalities and the fuzzy based weight assessment has enabled offering less spectral information loss as compared to other state of art techniques. The improvement in fused images is more visible in the tumorous region (bottom right corner).

**Figure 4:**
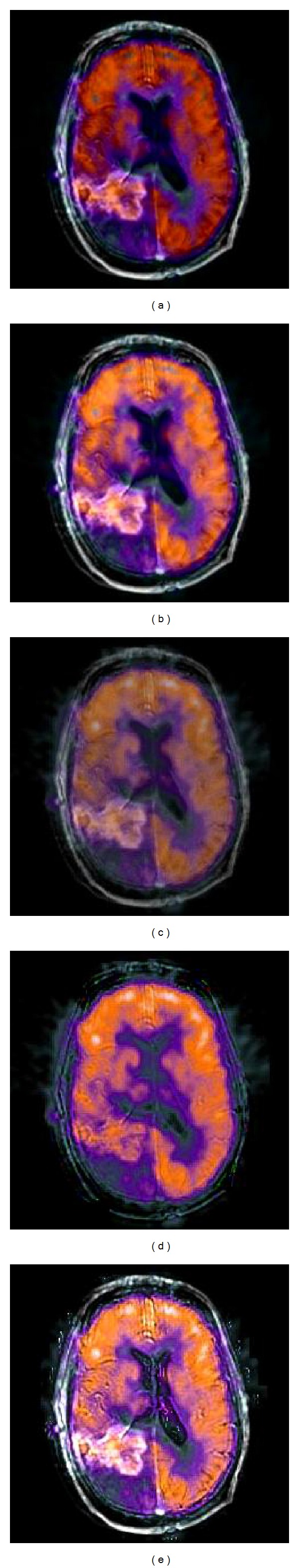
Image fusion results for grade IV astrocytoma images: (a) DWT [12]; (b) GIHS [6]; (c) GFF [33]; (d) IAWP [23]; (e) proposed technique.

Figure 4 shows fused images (of Grade IV astrocytoma) obtained by using different techniques. Similar improvement (as that of Figures 2(e) and 3(e)) can be observed in Figure 4(e). It is easy to conclude that the proposed scheme provides better visual quality compared to the existing schemes.

Table 1 shows the quantitative comparison of different fusion techniques. Note that a higher value of the metric represents better quality. The fused images obtained using proposed technique provide better quantitative results in terms of entropy [29], MI [29], SSIM [30], Xydeas and Petrovic [31], and Piella [32] metrics.

## 5. Conclusion

An image fusion technique for MRI and PET using local features and fuzzy logic is presented. The proposed scheme maximally combines the useful information present in MRI and PET images using image local features and fuzzy logic. Weights are assigned to different pixels for fusing low frequencies. Simulation results based on visual and quantitative analysis show that the proposed scheme produces significantly better results compared to state of art schemes.

## 6. Conflict of Interests

The authors declare that there is no conflict of interests regarding the publication of this paper.

## Figures and Tables

**Table 1:** Quantitative measures for fused PET-MRI images.

Scenario	Techniques	Entropy [29]	MI [29]	SSIM [30]	Xydeas and Petrovic [31]	Piella [32]
Normal brain	DWT [12]	5.403	1.6607	0.6083	0.4944	0.7558
	GIHS [6]	5.381	1.7017	0.7095	0.5362	0.8014
	GFF [33]	5.115	1.7479	0.6803	0.4825	0.6741
	IAWP [23]	5.152	1.7753	0.6735	0.3233	0.3331
	Proposed	5.738	1.7912	0.6788	0.5746	0.8469

Grade II astrocytoma	DWT [12]	3.4820	1.3817	0.7287	0.6495	0.8566
	GIHS [6]	3.4679	1.3848	0.8149	0.6227	0.8779
	GFF [33]	3.5558	1.3758	0.8120	0.6417	0.8561
	IAWP [23]	3.6351	1.3770	0.8018	0.3757	0.5405
	Proposed	3.5762	1.4292	0.8133	0.6674	0.9125

Grade IV astrocytoma	DWT [12]	5.4140	1.7487	0.6775	0.5727	0.8434
	GIHS [6]	5.7868	1.7084	0.6207	0.5697	0.8547
	GFF [33]	5.6628	1.7883	0.6819	0.5112	0.7917
	IAWP [23]	5.6831	1.8298	0.6718	0.3584	0.5642
	Proposed	5.8204	1.8683	0.6739	0.5885	0.8755
